# Diabetes MILES Youth–Australia: methods and sample characteristics of a national survey of the psychological aspects of living with type 1 diabetes in Australian youth and their parents

**DOI:** 10.1186/s40359-016-0149-9

**Published:** 2016-08-12

**Authors:** Virginia Hagger, Steven Trawley, Christel Hendrieckx, Jessica L. Browne, Fergus Cameron, Frans Pouwer, Timothy Skinner, Jane Speight

**Affiliations:** 1 Centre for Social and Early Emotional Development, School of Psychology, Deakin University, Geelong, VIC 3220 Australia; 2 The Australian Centre for Behavioural Research in Diabetes, Diabetes Victoria, 570 Elizabeth Street, Melbourne, 3000 Australia; 3 Department of Endocrinology and Diabetes and Centre for Hormone Research, Royal Children’s Hospital and Murdoch Children’s Research Institute, Melbourne, 3000 Australia; 4 Department of Medical and Clinical Psychology, Center of Research on Psychology in Somatic diseases (CoRPS) TSB, Tilburg University, Warandelaan 2, Tilburg, 5037 AB The Netherlands; 5 School of Psychological and Clinical Sciences, Charles Darwin University, Darwin, 0909 Australia

**Keywords:** Type 1 diabetes, Psychological well-being, National survey, Adolescents, Self-care, Quality of life, Diabetes distress, Depression

## Abstract

**Background:**

Type 1 diabetes is a complex and demanding condition, which places a substantial behavioural and psychological burden on young people and their families. Around one-third of adolescents with type 1 diabetes need mental health support. Parents of a child with type 1 diabetes are also at increased risk of psychological distress. A better understanding of the motivators, behaviours and psychological well-being of young people with diabetes and their parents will inform improvement of resources for supporting self-management and reducing the burden of diabetes. The Diabetes MILES (Management and Impact for Long-term Empowerment and Success) Youth–Australia Study is the first large-scale, national survey of the impact of diabetes on the psychosocial outcomes of Australian adolescents with type 1 diabetes and their parents.

**Methods/design:**

The survey was web-based to enable a large-scale, national survey to be undertaken. Recruitment involved multiple strategies: postal invitations; articles in consumer magazines; advertising in diabetes clinics; social media (e.g. Facebook, Twitter). Recruitment began in August 2014 and the survey was available online for approximately 8 weeks. A total of 781 young people (aged 10–19 years) with type 1 diabetes and 826 parents completed the survey. Both genders, all ages within the relevant range, and all Australian states and territories were represented, although compared to the general Australian population of youth with type 1 diabetes, respondents were from a relatively advantaged socioeconomic background.

**Discussion:**

The online survey format was a successful and economical approach for engaging young people with type 1 diabetes and their parents. This rich quantitative and qualitative dataset focuses not only on diabetes management and healthcare access but also on important psychosocial factors (e.g. social support, general emotional well-being, and diabetes distress). Analysis of the Diabetes MILES Youth–Australia Study data is ongoing, and will provide further insights into the psychosocial problems facing young people with type 1 diabetes and their parents. These will inform future research and support services to meet the needs of young Australians with type 1 diabetes and their families.

**Electronic supplementary material:**

The online version of this article (doi:10.1186/s40359-016-0149-9) contains supplementary material, which is available to authorized users.

## Background

Diabetes places substantial behavioural and psychological burden on young people and their families. Type 1 diabetes (T1DM) is the most common form among youth, and Australia has one of the highest incidences worldwide (24 per 100,000 aged 10–19 years) [1, 2]. In 2014, there were 9856 Australians aged between 10 and 19 years living with T1DM [3].

Managing T1DM is challenging at any age, and particularly so during adolescence. The transition from childhood into adulthood is characterised by significant physical, cognitive, social and emotional developments. These changes can affect diabetes management in several ways. Hormonal changes and changes in insulin sensitivity often lead to increased blood glucose levels [4]. Gaining body weight, more frequent among girls than boys with T1DM or peers without diabetes [5], can become a source of body dissatisfaction [6, 7]; and may be associated with weight control behaviours, including insulin restriction [8]. Performing diabetes self-care tasks requires cognitive maturity. If the young person is not ready to take on these responsibilities, but is expected to do so, this may lead to conflict (with family and health professionals) and disengagement from diabetes management.

Social changes during this transition are substantial. It is a period of gaining independence on the one hand, but still needing support from parents. Parental authority diminishes and peers become more influential [9]. The adolescent spends less time at home, reducing parental supervision of their diabetes self-care. Being with friends more often is accompanied by changes in eating behaviours (e.g. fast food), engaging in sexual relationships, in risk-taking behaviours (e.g. experimenting with alcohol, smoking, other drugs) [10]. Friends may be very supportive and caring of the adolescent with diabetes; but some may have a negative influence, leading to social pressure not to be “different” from their peers. All these changes can contribute to diabetes self-care being neglected [11], such as not checking blood glucose or skipping insulin doses, contributing further to sub-optimal blood glucose levels, thereby increasing the risk of complications [12].

Not surprisingly, these challenges during adolescence can compromise the young person’s emotional health and well-being [13, 14]. While most adjust well to living with T1DM, around one-third need mental health support [15, 16]. Compared with the general population, adolescents with T1DM experience more than double the rate of elevated depressive symptoms [17, 18]. Although less researched, diabetes distress also appears to be common, with over half of adolescents reporting at least one aspect of diabetes is a serious problem for them [19]. A review of studies in adults with T1DM found that 20–30 % experience elevated diabetes distress, and indicated an association between diabetes distress, less attention to self-care and high HbA1c [20]. However, among adolescents the prevalence of diabetes distress is unknown and the relationship between distress and diabetes management is inconsistent, and needs further investigation using age-appropriate measures [21]. Despite awareness of impaired emotional well-being among adolescents with T1DM, only a quarter of those who might benefit from psychological support actually receive it [18]. Moreover, unresolved mental health problems often carry into adulthood [22], so adolescence is an important stage for identifying problems and early intervention.

With regard to parental well-being and concerns, most studies to date have focused on parents of a young child with T1DM, but less is known about the parents of adolescents. Among mothers of an adolescent with T1DM, clinically-significant levels of depressive and anxiety symptoms have been reported (18–26 % and 13–55 % respectively) [23, 24]. Among fathers, up to 13 % have elevated depressive symptoms and 23 % have anxiety [23]. While the burden of diabetes care may be higher for parents of a young child, the stress of parenting is unlikely to decline for parents as their child becomes an adolescent, thus depressive and anxiety symptoms may not lessen as their child grows up [25, 26]. Furthermore, the emotional burden for parents does not diminish with longer duration of living with diabetes [27], with diabetes distress apparent among parents of children and adolescents with T1DM [28, 29].

Hypoglycaemia is a source of worry and distress for parents [27, 30]. Parents who are very worried about hypoglycaemia check their child’s blood glucose more frequently [30]. However parental worry about hypoglycaemia is also associated with elevated HbA_1c_ among children, suggesting that parents may overcompensate in their attempts to avoid hypoglycaemia (e.g. by reducing insulin doses) [30]. At the same time, worry about high blood glucose and future complications is a major concern for parents [29]. These concerns can lead to frustration and family conflict if the young person assumes responsibility for self-management and their attention to this wanes [31]. Furthermore, unresolved family conflict [32] and impaired parental mental health [33] has been associated with adverse psychological and diabetes-related health outcomes among youth with T1DM.

To date, no national survey has examined the psychosocial outcomes of Australian adolescents and parents living with diabetes. Thus, a better understanding of the psychological well-being, behaviours and support needs of Australian youth with diabetes and their parents is needed, to inform improvement of services and facilities for supporting self-management and reducing the burden of diabetes.

The Diabetes MILES (Management and Impact for Long-term Empowerment and Success) Study is an international collaborative co-led by Professor Jane Speight (Diabetes MILES–Australia) and Professor Frans Pouwer (Diabetes MILES–The Netherlands). The aim is to further promote understanding and awareness of the psychological and behavioural aspects of living with diabetes by conducting a series of national surveys of people with type 1 or type 2 diabetes in various countries (including Diabetes MILES–The Netherlands and Diabetes MILES–Flanders). In 2011, Diabetes MILES–Australia was the largest survey ever conducted of the psychosocial and behavioural aspects of living with type 1 or type 2 diabetes among Australian adults [34]. Completed by 3338 adults, this national survey provided important insights into how Australians manage their diabetes, the support they receive and the impact of the condition on their psychological well-being and quality of life.

The Diabetes MILES Youth–Australia Study (MILES Youth) provides the opportunity to address the research questions discussed above; in particular, to explore how diabetes distress is related to other psychological problems (e.g. depressive symptoms), and to diabetes management, as well as family and health professional support. Few data are available about parents of adolescents with T1DM, their own emotional well-being, their concerns about their child’s diabetes, or the impact of these factors on their child’s diabetes management, and almost none in the Australian context.

### Aim

The aim of the MILES Youth Study was to investigate psychological and behavioural issues in a large-scale, national sample of young people (aged 10–19 years) with T1DM and their parents. In particular, the study focuses on:

- The extent to which young people with diabetes are actively managing their condition, engaging with recommended self-care strategies and healthcare providers;
- The perceived impact of living with diabetes (including its management and acute complications) on quality of life and emotional well-being, specifically assessing diabetes distress, anxiety and depression;
- The extent to which young people with diabetes: (a) feel empowered to manage their condition, (b) perceive that their health professionals are supportive, (c) have access to and have accessed appropriate healthcare resources in the past year;
- Aspects of positive mental health associated with ‘living well’ with diabetes, as well as identifying personal strengths and support from peers, family and healthcare professionals that mediate optimal outcomes.

The findings will be disseminated to raise awareness of the psychosocial well-being and unmet needs of Australian adolescents living with T1DM and of their parents, and to inform recommendations for the resources and services that would be of benefit.

## Methods/design

### Establishment and role of the reference groups and funding body

A MILES Youth Study reference group was established comprising 12 academics and/or clinicians with relevant expertise, including paediatric endocrinologists, diabetes educators, clinical and health psychologists–four were based outside Australia. The purpose of the reference group was to advise on survey concepts and research questions and their operationalisation (including validated measures and discrete variables). The reference group members will continue to collaborate on publications and dissemination of the study results.

The MILES Youth study was commissioned and funded by the National Diabetes Services Scheme (NDSS) Young People and Diabetes (YPD) National Development Programme. The NDSS is an initiative of the Australian Government, administered by Diabetes Australia. The NDSS YPD Expert Reference Group, comprising clinicians, academics, young adults with T1DM and administrators, reviewed the survey to ensure the content was relevant to young people with T1DM and their parents, the NDSS and the Australian context. The funding body played no further role in determining research questions, analysing data or interpreting findings.

### Phase 1: survey design and selection of measures

Informed by the approach of the previous Diabetes MILES Australia study (for adults) [34], the MILES Youth survey was developed by following three key steps:

#### Defining the survey topics

MILES Youth reference group members were interviewed to identify current evidence gaps and survey concepts related to the aims of the study. Based on these consultations, the survey concepts were selected by the research team for both adolescents and their parents (Table 1).

**Table 1:** Concepts and measures (youth and parent surveys)

Concept	Measure or variable	Number of items		
		Youth version (age group: years)		Parent version
		10–12	13–19	
About You				
Demographics	Age, gender, family composition, language, education, employment	12	13	12
	Health insurance, financial status			3
Stressful life events	Items adapted from Recent Life Events Questionnaire [48]			14
Diabetes history	Diabetes type, treatment, duration, family history	4	4	8
Mood				
General quality of life	Item from MIND Youth Questionnaire (MY-Q) [7] derived from Diabetes Quality of Life for Youth–Short Form [49]	1	1	
Well-being	WHO-5 Well-being Scale [7, 50–52]	5	5	5
Depressive symptoms	Patient Health Questionnaire for Adolescents (PHQ-A) [53, 54]		8	
Anxiety	Generalised Anxiety Disorder Scale (GAD-7) [55]		7	7
Feelings About Diabetes				
Diabetes distress	Problem Areas in Diabetes–Teen version (PAID-T) [19]		26	
	Problem Areas in Diabetes–Parent of Teens version (P-PAID-T) [29]			26
Family conflict	Items from MY-Q [7] derived from the Diabetes Family Conflict Scale [56]	2	2	
Responsibility for diabetes management	Items from MY-Q [7]	2	2	
	Items modified from the Diabetes Family Responsibility Questionnaire [57]			5
Health & Health Checks				
General health	Other health conditions	1	1	1
	Weight, height	2	2	
Perceived health^a^	Self-rated health		1	
Diabetic ketoacidosis (DKA)	Incidence of diabetic ketoacidosis		1	3
Worry about hyperglycaemia	Items from the Hyperglycaemia Avoidance Scale [58]			3
Diabetes Care				
Blood glucose monitoring^a^	Self-reported frequency of self-monitoring of blood glucose (SMBG)	2	2	2
HbA1c	Self-reported HbA1c	2	2	4
Insulin management^a^	Insulin dose frequency	1	1	1
	Insulin forgetting & omitting adapted from MY-Q [7] and Adolescent Diabetes Needs Assessment Tool (ADNAT) [59]		3	
Hypoglycaemia				
Hypoglycaemia frequency	Items adapted from Hypoglycaemia Awareness Questionnaire (HypoA-Q) [60]		6	7
Hypoglycaemia awareness	Gold score [61]		1	1
	Item adapted from HypoA-Q [60]		1	1
Fear of hypoglycaemia	Hypoglycaemia Fear Survey for parents (PHFS) and children (CHFS) [62]		25	25
Technical/medical support^a^	Technology and hypoglycaemia			2
	Communication with doctor about hypoglycaemia			2
Eating Habits				
Diabetes-specific eating behaviours	Diabetes Eating Problem Survey-Revised (DEPS-R) [63]		16	
	Binge eating frequency adapted from MY-Q [7]		1	
Body image	Gender-specific body image silhouettes from BMI-based Silhouette Matching Test (BMI-SMT) [64, 65]		3	
Health Care Team				
Patient-centred communication (PCC)	PCC subscale of the Health Care Climate Questionnaire [66, 67]		5	5
Treatment satisfaction	Items from MY-Q [7]; derived from the Diabetes Treatment Satisfaction Questionnaire (DTSQs) [68]	3	3	3
Health professional support^a^	Free text: (what I wish health professionals knew…)	1	1	1
Transition	Items adapted from Online Transition to Adulthood Surveys for Youth with Chronic Illness [69]			3
Diabetes care^a^	Child’s diabetes healthcare providers & attendance			7
Support to Manage Diabetes				
Resilience	Diabetes Strengths and Resilience Measure for Adolescents (DSTAR-Teen) [70]	12	12	
Self-efficacy	Maternal Self-Efficacy for Diabetes Management Scale [71]			17
Social support^a^	Free text: (what I wish friends/teachers/general public knew about diabetes)	2	2	3
	(what friends/teachers do to help)	2	2	
	(what would make it easier for you/your child…)			2
Parental support^a^	2 free text: (what I wish my parents knew about diabetes; what my parent do to help me..)	2	2	
NDSS support	Free text		1	1
Technology^a^	Use of ‘apps’ for diabetes management	5	5	-
Final comments	Free text	1	1	1
Unique ID	Child’s NDSS Number	1	1	1

^a^Designed by the research team in the absence of relevant and suitable standardised measures

#### Identification and assessment

For each concept, a search was undertaken for questionnaires appropriate for use in adolescents (aged 10–19 years) or parents/adults. Each questionnaire was considered with regard to its content and construct validity and internal consistency reliability, length, and previous use within an adolescent and/or diabetes-specific population. If relevant and appropriate validated measures were not identified, study-specific questions were created relating to these themes. Linguistic and literacy considerations were assessed, both by members of the research team and through pilot testing and cognitive debriefing (see below) with young people living with diabetes and their parents. This process resulted in an item bank that was reviewed by the reference groups during the subsequent consultation phase (see below).

#### Consultation

Reference group members provided feedback regarding suitability of the item bank. Questionnaires or individual items that were considered inappropriate for the purposes of the study were removed and alternatives suggested. This process continued for several iterations until no further modifications were suggested by the reference group. The reference groups expressed some concerns about survey length (for all age groups) and the sensitivity of some issues (e.g. eating behaviours, depression, diabetes distress) for younger respondents. In addition, they were concerned about asking adolescents about suicidal ideation (item 9 of the PHQA-9).

### Phase 2: pilot study and cognitive debriefing

The aim of the pilot study was to ensure that the survey content was acceptable, relevant and suitable for young people with T1DM and their parents, and to determine how long it took for participants to complete the surveys.

#### Recruitment

Young people (aged 10–19 years) with T1DM and their parents were eligible. They were invited to take part in the pilot study via letter, social media or electronic newsletter distributed to members of Diabetes Victoria, the peak body for people with diabetes in Victoria. Potential participants contacted the research team by telephone or email, and were then sent (by email or post) a copy of the plain language statement, and a consent form to sign.

#### Procedure

Upon consent, volunteers were emailed a link to the online survey and posted a hard copy of the questionnaire to review. They were asked to complete the questionnaire online no more than one day prior to the interview and note their thoughts about the questions, the response options and instructions on the hard copy. Interviews were audio-recorded to enable reflection upon responses. During the interview, participants were asked structured questions about the survey’s suitability and relevance, the layout and length, the language and how easy it was to understand, and website usability. Interviews ranged from 15 to 60 minutes with adolescents and 20–35 min with parents.

Cognitive debriefing interviews were conducted with 13 people living in Victoria (12 via telephone, and one face-to-face): eight young people with T1DM (4 (50 %) girls; three aged 11–12 years and five aged 16–18 years; all in full-time school education, except one boy) and five mothers of children with T1DM. Four of the mothers were parents of the participating youths and all had completed high school or tertiary education.

Young people aged 11–12 years reported taking 15–20 min to complete the survey, whereas the completion time for older adolescents (who received the longer questionnaire) ranged from 20 to 60 min. The time reported by parents to read and complete the parent survey ranged from 20 to 35 min. Overall, young people and their parents were positive in their feedback about the survey, indicating they considered the topics relevant and meaningful and the language appropriate. Participants requested that a few terms should be defined and instructions shortened. Two adolescents stated that the survey was too long.

### Phase 3: finalising survey content and study materials

Several modifications were made to the survey in response to feedback received, including removing items to reduce length, simplifying instructions, providing definitions and rearranging the order in which items were presented (e.g. generic before diabetes-specific items; open-ended questions and personal information towards the end). In response to concerns expressed by the reference groups (Phase 1) and by parents (Phase 2), items relating to eating disorders were removed from the youth survey, body image questions were removed for younger children and the cut-off for the younger age group was raised to 12 years. Three new items concerning diabetic ketoacidosis (DKA) were added to the parent survey. The final suite of concepts investigated and the measures used in each version of the survey are listed in Table 1. Approval to use the various measures, and a license (where required) was obtained from scale developers/copyright holders. Three versions of the survey were approved by the Deakin University Human Research Ethics Committee to be suitable for:

(i) young people aged 10–12 years (63 items)
(ii) young people aged 13–19 years (169 items)
(iii) parents of young people aged 10–19 years (176 items)

Additional file 1 provides a description of the scales used in the Diabetes MILES Youth Study.

### Phase 4: data collection–national online survey

#### Eligibility and recruitment

People were eligible to participate if they met the following inclusion criteria:

- They were a young person (aged 10–19 years of age inclusive), with diagnosed T1DM; or if they were the parent of such a person
- They had previously consented to the NDSS contacting them for research purposes (60 % of registrants (or their parents if under 18 years) had done so)
- They completed at least the ‘mood’ module of survey questions, considered to be the core dataset.

The purpose of the NDSS is to provide subsidised products (i.e. needles, insulin pump consumables, blood glucose test strips), information and support services for Australians diagnosed with diabetes. All young people with T1DM are registered with the scheme (*N* = 9856 aged 10–19 years at the time of the survey) [NDSS, Personal Communication, October 2014]. Invitation letters were posted to all NDSS registrants (or their parents, if the registrant was less than 18 years old) meeting the first two of the above criteria. Thus, recruitment letters were distributed to 5928 eligible NDSS registrants or their parents, inviting them to complete the online survey (or to request a paper version if preferred; no such requests were received). The survey was also advertised via flyers in diabetes clinics, social media postings, at diabetes events, and notices in relevant publications (e.g. Diabetes Australia state and territory member magazines and e-newsletters). All recruitment material indicated that completing the online survey would provide an opportunity for the respondent to be entered into a prize draw to win a tablet computer. The survey was open for a period of 8 weeks from August to October 2014.

A response rate of approximately 18 % (*N* = 1000) was anticipated, based on the response to the adult Diabetes MILES survey [34], which would offer adequate power for multivariate and subgroup analyses.

#### Procedure

All surveys were administered online using Qualtrics^TM^, a secure, online survey-hosting platform. Registrants and parents were directed to a webpage that provided additional information (plain language description) about the nature of the study. They were requested to give their consent to participate before proceeding to the survey. All respondents were asked to provide the young person’s NDSS registration number (a unique identifier), for the sole purpose of matching parent and child survey responses to enable dyad analyses. The researchers did not have access to the NDSS database, thus could not identify respondents from their NDSS registration number. At the end of the survey, all respondents were invited to provide their contact details: a) to enable entry into the prize draw, and/or b) to express their willingness to be contacted for further research. These contact details were entered into a separate database not linked to the main survey to ensure the survey dataset remained de-identified. It was not mandatory to provide contact details.

### Phase 5: data handling and analyses

All survey responses, both complete and incomplete, were logged by the Qualtrics^TM^ survey platform and downloaded at survey close (October 2014) into data files for analysis in the Statistical Package for the Social Sciences (SPSS) (IBM SPSS Statistics for Windows, Version 22.0. Armonk, NY: IBM Corp). Descriptive statistics will be reported as counts and percentages (N (%)) for categorical variables and mean ± standard deviation (or medians and ranges as appropriate for data distributions) for continuous variables. Differences between groups will be analysed using *χ*^2^ tests for categorical data and independent samples t-tests or ANOVAs for continuous variables. More advanced analyses (e.g. multiple regression, factor analysis) will be applied as appropriate to specific research questions and will be reported in subsequent papers. The qualitative data will be analysed using thematic and/or content analyses, as appropriate to particular research questions.

#### Response rates and exclusions

During the 8 weeks the survey was available, 934 and 1050 responses were collected in the young persons and parent surveys respectively. Consistent with the inclusion criteria, respondents’ completed surveys were excluded if:

- they did not provide the youth’s age or the age did not meet the inclusion criteria (youth 8 %, *n* = 79; parents 4 %, *n* = 47);
- they did not provide the youth’s diabetes type (youth 2 %, *n* = 15; parents 15 %, *n* = 161);
- the youth did not have T1DM, i.e. reported type 2 diabetes or an “other type”, e.g. Maturity Onset Diabetes of the Young (youth <1 %, *n* = 8; parents <1 %, *n* = 5);
- did not attempt the mood questions (youth 4 %, *n* = 39; parents <1 %, *n* = 3), since this was considered the core dataset.

The final samples included:

- *N* = 781 young people (aged 10–19 years) with T1DM;
- *N* = 826 parents of young people with T1DM.

Of these, 89 % (*n* = 698) youth and 89 % (*n* = 736) parents answered all questions in their survey version. In total, *N* = 258 youth/parent dyads could be identified by matching the young person’s NDSS number to the NDSS number reported by a parent.

#### Sample characteristics

Respondents were from all states and territories, including metropolitan, regional and remote areas of Australia (Table 2). The representativeness of the sample was determined by comparing youth respondents on key characteristics, i.e. age, gender, socio-economic status (SES) and residential location, to NDSS registrants in the corresponding age group (Table 2). The Australian Bureau of Statistics (ABS) Index of Relative Socio-Economic Advantage/Disadvantage (IRSAD) [35] was used to index SES. This measure summarises census data related to both advantage and disadvantage (e.g., income, education and unemployment) within a postcode area. An IRSAD decile code was computed for each respondent using the postcode they provided. Residential area was classified using the ABS remoteness areas structure [36]. Almost half the respondents were from a high socio-economic background and resided in a metropolitan area (Table 2). Finally, more than a quarter (30 %, *n* = 232) of all respondents used a mobile device (e.g., smartphone or tablet) to complete the survey (youth 30 %, *n* = 232; parents 29 %, *n* = 243).

**Table 2:** Demographic and clinical characteristics for youth with type 1 diabetes (*N* = 781) and parents (*N* = 826)

	Youth (*N* = 781)	Parents (*N* = 826)	NDSS Registrants aged 10–19 years (*N* = 9856)^a^
Gender–female	474 (61)	727 (88)	4672 (47)
Child’s gender–female	-	384 (47)	-
Age–years	14 ± 3	46 ± 6	16 ± 3
Child’s age–years		14 ± 3	
Youth/child’s age group–years			
10–12	230 (29)	285 (35)	2078 (21)
13–15	277 (35)	292 (35)	2986 (30)
16–17	153 (20)	155 (19)	2312 (23)
18–19	121 (15)	94 (11)	2480 (25)
State/Territory			
New South Wales	211 (27)	205 (25)	2980 (30)
Victoria	184 (24)	248 (30)	2512 (25)
Queensland	182 (23)	162 (20)	2139 (22)
Western Australia	87 (11)	92 (11)	985 (10)
South Australia	68 (9)	70 (8)	743 (8)
Tasmania	33 (4)	21 (2)	260 (3)
Australian Capital Territory	15 (2)	23 (3)	179 (2)
Northern Territory	1 (<1)	4 (<1)	58 (<1)
Geographical area	(*N* = 755)	(*N* = 810)	
Major cities	517 (68)	546 (67)	6692 (69)
Inner regional	168 (22)	192 (24)	2188 (22)
Outer regional & remote	70 (9)	72 (9)	876 (9)
Socio-economic status–IRSAD	(*N* = 754)	(*N* = 810)	
Low (1–3)	130 (17)	121 (15)	2210 (23)
Medium (4–7)	284 (38)	319 (39)	4069 (42)
High (8–10)	340 (45)	370 (46)	3468 (36)
Cultural/ethnic background			
Aboriginal or Torres Strait Islander	14 (2)	9 (1)	188 (2) (*N* = 8595)
Country of birth–Australia	715 (92)	659 (80)	5447 (87) (*N* = 6280)
Main language spoken at home–English	759 (97)	808 (98)	-
Child lives with	(*N* = 758)	-	-
2 parents (biological or adoptive)	624 (82)		
2 parents–one a step-parent	50 (7)		
Single parent family	85 (11)		
Youth/child’s diabetes			
Age at diagnosis (years)	9 ± 4	8 ± 4	9 ± 4
Diabetes duration (years)	6 ± 4 (0–18)	6 ± 4 (0–16)	6 ± 4 (0–19)
Treatment regimen–CSII	409 (52)	436 (53)	4084 (41)^b^
Self-reported HbA1c–mmol/mol (%) (*N* = 650)	65 ± 18 (8.1 ± 1.6 %)	64 ± 16 (8.0 ± 1.4)	-
Occupation	(*N* = 773)	(*N* = 739)	-
School student	676 (87)	0	
Tertiary student (university)	51 (6)	0	
Employed/self-employed, full/part time	22 (3)	570 (77)	
Apprenticeship or trade training	13 (2)	0	
Unemployed/Looking for work	11 (1)	22 (3)	
Homemaker/Carer/Volunteer	1 (<1)	130 (18)	
Other	11 (1)	17 (2)	
Parents’ marital status	-	(*N* = 823)	-
Married		651 (79)	
De facto (living together)		57 (7)	
Relationship (living apart)		9 (1)	
Single		11 (1)	
Separated/divorced		84 (10)	
Widowed		11 (1)	
Parents’ highest level of education	-	(*N* = 740)	-
≤year 10		98 (13)	
Completed year 12		103 (14)	
Trade/diploma		222 (30)	
University		317 (43)	
Annual household income ($)	-	(*N* = 711)	-
Up to 20,000		14 (2)	
20,001–40,000		47 (7)	
40,001–60,000		66 (9)	
60,001–80,000		87 (12)	
80,001–100,000		126 (18)	
>100,001		264 (37)	
Prefer not to answer		107 (15)	

Unless otherwise stated, data are n (%) or mean ± SD (range)

Total N reported in this table not always consistent with total sample size due to missing data on some items

*IRSAD* Index of Relative Social Advantage and Disadvantage, *CSII* Continuous subcutaneous insulin infusion

^a^Total number of NDSS Registrants with type 1 diabetes aged 10–19 years at November 2014

^b^As at June 2014

##### Young people with type 1 diabetes

Of the 781 young people who responded, the mean age was 14 ± 3 years (range 10–19) and 61 % (*n* = 474) were girls (Table 2). The majority (92 %, *n* = 715) were born in Australia and, for 97 % (*n* = 759), English was their primary language. Fourteen respondents (2 %) reported being of Aboriginal and/or Torres Strait Islander descent. Eighty percent (*n* = 624) of young people lived with both parents. Mean diabetes duration was 6 ± 4 years (range 0–18). Nineteen percent (*n* = 149) had been diagnosed with T1DM for less than 1 year. Fifty-two percent (*n* = 409) managed their T1DM using an insulin pump and 38 % of respondents had self-reported a glycosylated haemoglobin (HbA1c; average blood glucose over the past 8–12 weeks) within recommended target range (<58 mmol/mol; <7.5 %) [37].

##### Parents of young people with type 1 diabetes

Of the 826 parent respondents, their mean age was 46 ± 6 years (range 30–73), and 88 % (*n* = 727) were mothers (Table 2). While 20 % (*n* = 167) of parents were not born in Australia, only 2 % (*n* = 18) did not speak English at home. A very small number of parents (1 %; *n* = 9) reported being of Aboriginal and/or Torres Strait Islander descent. The majority (86 %, *n* = 708) were married or in a de facto relationship, 93 % (*n* = 686) of parents or their partners were in paid employment, and 37 % (*n* = 264) had a total annual household income above $100,000. The characteristics of their children were similar to the youth respondents; mean age 14 ± 3 years; mean duration of diabetes 6 ± 4 years, 53 % (*n* = 436) used an insulin pump and self-reported mean HbA1c 64 ± 16 mmol/mol (8.0 ± 1.4 %), although fewer were female (*n* = 384, 47 %) and only 7 % (*n* = 56) had been diagnosed in the past year.

##### Youth/parent dyads

Among the youth, boys and girls were almost equally spilt in the dyad dataset (female 53 %, *n* = 136). Compared to the overall sample, the mean age of the adolescents was lower (13 ± 2 years). Accordingly, the duration of diabetes was shorter (5 ± 4 years). The dyad sample did not differ from the overall sample on other demographic characteristics: born in Australia (92 %, *n* = 238); metropolitan location (64 %, *n* = 166); socio-economic status (IRSAD: 17 %, *n* = 43 low; 38 %, *n* = 97 medium; 45 %, *n* = 117 high SES); single-parent family 8 % (*n* = 21).

#### Qualitative responses

Open-ended questions with space for free-text responses offered respondents the opportunity to communicate their experience of living with diabetes in their own words and their feedback on the survey (Table 1). Most participants responded to at least one of the open questions; only 22 (3 %) young people and 74 (9 %) parents did not respond to any.

## Discussion

The MILES Youth Study is the first large-scale, national survey of young Australians living with T1DM (and their parents) focused not only on diabetes management and healthcare access but also on psychosocial outcomes. In total, 781 young people with T1DM completed the survey, which represents 13 % of the 5928 NDSS registrants with T1DM invited to take part. In addition, 826 parents of young people with T1DM aged 10–19 years responded to the survey. A sub-sample comprising of 258 parent/child dyads were matched using the youths’ NDSS registration number.

The responses to the MILES Youth survey will provide insights into the main concerns and worries about living with T1DM for Australian adolescents. While previous studies suggest that most young people are likely to be coping well with diabetes and have optimal emotional well-being, the survey results will provide an indication of how many are experiencing elevated depressive and anxiety symptoms, elevated diabetes distress, and what is causing the distress. We will also gain a better understanding of how these negative moods and feelings are related to individual and family characteristics, and in particular, whether there are differences in the expressed emotions and self-care behaviours of older and younger adolescents or girls and boys.

The MILES Youth dataset allows us to identify risk and resilience factors for young people and their parents. For example, hypoglycemia is a common acute complication of insulin treatment, yet in this age group (and in parents) in Australia, we know little about the frequency and severity of hypoglycaemia, impaired awareness of hypoglycaemia and the impact of hypoglycaemia on emotional well-being.

The feedback of young people related to the perceived support they receive from parents, teachers, friends and healthcare professionals, and how this helps them in managing their diabetes, will inform the development of services and resources to better support young people with T1DM (and their families). For example, greater awareness of the needs and concerns of adolescents (and their parents) as they approach adulthood and independence will assist diabetes services to improve the process of transition from paediatric to adult healthcare and reduce the number of young people ‘lost in transition’ [38]. The MILES Youth findings will also be used to raise awareness amongst clinicians and policy makers of the psychological and behavioural challenges that many young people and their families face and the current gaps in services to address these needs, and to advocate for resources and better access to care.

To our knowledge, MILES Youth is the first national study with matched parent and child responses regarding living with T1DM. Analysis of the parent/child dyads will progress our understanding of family-related factors, and the interaction between parental well-being and support and youths’ self-care behaviours and psychological well-being. In-depth analysis of the dataset is ongoing, and peer-reviewed publications are planned. Half of the respondents (55 %) indicated their interest in future studies. Using the NDSS number to link survey responses, and with appropriate ethics approval, future data collections could enable a longitudinal study to follow these young people into adulthood, to investigate the long-term impact of their behaviours and well-being on future outcomes.

### Strengths and limitations

Qualitative feedback from participants and the high proportion of complete datasets (89 %) indicates the survey was relevant and addressed important issues for young people with diabetes and their parents. Young people found the language and topics resonated with their experience of living with diabetes. The online format was a successful and economical approach for engaging young people with T1DM and their parents. Around one in four respondents used a mobile device to complete the online survey, suggesting the importance of mobile-friendly platforms when designing future online surveys and initiatives.

The proportion of NDSS registrants who participated in the MILES Youth study was generally equivalent by state, with the exception of Northern Territory, where participation was very low, most likely related to relative socio-economic disadvantage [39]. The majority of respondents were living in metropolitan areas in New South Wales, Queensland and Victoria, which reflects the geographic distribution of NDSS youth registrants (Table 2), and the distribution of the broader Australian population. Among youth respondents, both genders and all age ranges were well-represented, although there was an over-representation of girls and younger registrants.

Despite the fact that only one in three of the parent and child respondents were identified as being from the same family (*N* = 258 dyads matched by NDSS registration number), parent responses were remarkably consistent with those of young people for the corresponding survey items, e.g., participant demographics, duration of diabetes, treatment type.

The limitations of the study include self-selection bias. Invitations were sent to all those NDSS registrants who had consented to take part in research, which constitutes 60 % of registrants. Furthermore, participants were those who volunteered to take part, thus the sample may not be fully representative of the broader population of young people with diabetes and their families. The survey was available only in English, which is likely to have prevented some people from completing the survey. Not having access to a computer or the internet may also have precluded some people from taking part. However, in 2011, it was estimated that at least 79 % of Australian households have internet access [40] and no-one requested a hard copy survey, even though it was explicitly advertised as being available.

Limitations also exist in terms of the representativeness of the sample. Based on the IRSAD Index, respondents were from a relatively advantaged socioeconomic background compared to the total NDSS population aged 10–19 years. Thirteen percent of youth lived with one parent, which is fewer than the national average (22 %) for single-parent families (for children aged under 18 years) [41]. Insulin pump use was higher among MILES Youth respondents than the Australian average for this age group (53 % vs 41 % respectively; Table 2) most likely reflecting SES, since pumps are more accessible for Australians with private health insurance and those who can afford to pay the ‘out of pocket’ expenses for the hardware and consumables. MILES Youth respondents more frequently self-reported an HbA1c within target (<58 mmol/mol; <7.5 %) than the average for Australian paediatric diabetes centres (38 % versus 27 %) [42], although the mean HbA1c was only slightly lower than the average reported for 21 international centres in 2005 (8.0 vs 8.2 %) [43]. Nevertheless, HbA1c was self-reported, thus we acknowledge it may be subject to recall and social-desirability bias.

These indicators suggest that survey respondents may have better health literacy and access to healthcare services than young people with T1DM generally. Selection bias towards socially-advantaged families has been reported previously in a web-based study and was found to under-estimate the prevalence of psychopathology [44]. However, this bias is less likely to affect the relationships among study variables, which will be a key focus of our inferential data analyses. The overall survey response rate of 13 % was low, but cannot be compared with other studies as few in this younger age group have recruited in a comparable manner or have reported their response rate.

Finally, we acknowledge that we originally designed a survey version to be suitable for, and attempted to recruit into the study, NDSS registrants with type 2 diabetes and their parents (*N* = 417; 56 % of young people aged 10–19 with type 2 diabetes registered with the NDSS). However, none took part in the pilot and only 11 adolescents with type 2 diabetes and 8 parents responded to the survey: too few to analyse and report. Given the increasing prevalence of type 2 diabetes in younger people [45], the low response was disappointing but not unexpected, particularly given that many are likely to be experiencing socioeconomic disadvantage and/or are from culturally and linguistically diverse backgrounds (including Aboriginal and Torres Strait Islander communities) [46]. Based on previous research [47], families living in disadvantaged circumstances are likely to have higher rates of distress, impaired well-being and less access to healthcare. Thus, investigating and raising awareness of the unmet needs of young people with type 2 diabetes and their parents remains a high priority, although it is evident that other strategies are needed to reach them. Direct approaches via diabetes clinics, general practitioners or community groups may improve engagement with these families.

## Conclusions

The MILES Youth Study is the first large-scale, national survey of young Australians living with T1DM (and their parents) focused not only on diabetes management and healthcare access but also on a broad range of important psychosocial factors (e.g., general emotional well-being, diabetes distress, social support and quality of life) implicated in living well with this chronic condition. The study also represents the first step towards establishing a longitudinal program of research focused on the unmet needs of this group. Subsequent publications will report in-depth analyses of this rich quantitative and qualitative dataset to inform future research and support services to meet the needs of young people with T1DM and their families.

## Abbreviations

DKA, diabetic ketoacidosis; MILES, Management and Impact for Long-term Empowerment and Success; NDSS, National Diabetes Services Scheme; T1DM, type 1 diabetes mellitus; YPD, Young People and Diabetes (YPD)

## Additional file

Additional file 1: Description of scales used in the Diabetes MILES Youth Study. (PDF 201 kb)
