# Supplementary material for: Diabetes MILES Youth–Australia: methods and sample characteristics of a national survey of the psychological aspects of living with type 1 diabetes in Australian youth and their parents
Source: BMC Psychol. 2016 Aug 12;4:42. doi: 10.1186/s40359-016-0149-9 (PMC4983064; doi:10.1186/s40359-016-0149-9)
Supplement: Additional file 1: — Description of scales used in the Diabetes MILES Youth Study. (PDF 201 kb) [file 40359_2016_149_MOESM1_ESM.pdf]

Additional File 1: Description of scales used in the Diabetes MILES Youth Study

Name	Description
BMI-based Silhouette Matching Test (BMI-SMT) [64, 65]	The BMI-based-Silhouette Matching Test (BMI-SMT) uses gender-specific silhouette figures with reference points on a 27-point scale. Each point corresponds with a BMI score ranging between 14 to 40. The sets of silhouettes are presented three times and respondents are asked to choose a point on the scale that is (1) 'closest to the size you are now' (current); (2) 'closest to the size you would like to be' (ideal); (3) 'closest to the size you would be without diabetes' (a study-specific item). Body dissatisfaction is calculated by subtracting the ideal size score from the current size score.
Diabetes Eating Problem Survey-Revised (DEPS-R) [63]	A 16-item, diabetes-specific measure of disordered eating. Items are rated on a 6-point scale (0= Never; 5=Always). Item scores are summed, with total scores ranging from 0 to 80, and higher scores indicating more disordered eating behaviours.
Diabetes Family Responsibility Questionnaire (DFRQ) [57]	Five items from the DFRQ were used to identify parental perceptions about how diabetes self-care responsibilities are shared within the family. For each task parents choose: (i) child is mainly responsible, (ii) responsibility is shared, or (iii) the parent is mainly responsible. Items are analysed individually.
Diabetes Strengths and Resilience Measure for Adolescents (DSTAR-Teen) [70]	A 12-item self-report measure of adaptive attitudes and behaviours related to living with T1D. There are slight wording variations between the 9-13 years and 14-18 years versions that were used for the 10-12 year and 13-19 year age groups of the MILES Youth surveys respectively. Items are rated on a 5-point scale (0=Never; 4=Almost always). Item scores are summed (range 0-48), with higher scores indicating greater resilience.
Generalised Anxiety Disorder Scale (GAD-7) [55]	A 7-item scale that measures generalised anxiety symptoms. Respondents rate the frequency of anxiety symptoms over the past two weeks on a 4-point scale (0=Not at all, 3=Nearly every day). Item scores are summed to form a total score (range 0-21). Severity of anxiety symptoms is assessed using cut-off scores of 5, 10 and 15 representing mild, moderate, and severe anxiety symptoms respectively.
Gold score [61]	The single-item Gold score measures awareness of hypoglycaemic symptoms. Using a 7-point scale, respondents rate the extent to which they know when their 'hypos' are commencing (1=Always aware; 7=Never aware). A score of 4 or more implies impaired awareness.
Hypoglycaemia Fear Survey for parents (PHFS) and children (CHFS) [62]	The 25-item child and parent questionnaires adapted from the HFS-II assess childrens' and parents' worries (15-item subscale) and behaviours (10-item subscale) related to hypoglycaemia. Items are rated on a 5-point scale (0=Never; 4=Almost always). Subscale and total scores are obtained by summing the items for the worry subscale (range 0–60), the behaviour subscale (range 0–40) and the total score (range 0–100). Higher scores indicate greater fear of hypoglycaemia.
Maternal Self-Efficacy for Diabetes Management Scale (MSEDSM) [71]	A 17-item scale that assesses perceived parental confidence with specific diabetes management tasks. Each item is rated on a 5-point scale (1=Not confident at all; 5=Very confident without help). Scores are averaged across items with higher mean scores indicating greater self-efficacy.

Monitoring Individual Needs in Diabetes Youth Questionnaire (MY-Q) [7]	8 items from the MY-Q assess general QoL (1 item), diabetes responsibility (2 items) and family conflict (2 items), and treatment satisfaction (3 items). Items are rated (QoL, 0=Worst possible life; 10=Best possible life); diabetes responsibility and family conflict (1=All the time; 5=Never) and treatment satisfaction (1=Not at all happy; 5=Extremely happy); Treatment satisfaction for parents was rated 1=Very dissatisfied; 5=Very satisfied. Individual item scores are analysed, with higher scores indicating greater satisfaction/less conflict and treatment burden.
Patient-Centred Communication (PCC) [66, 67]	A 5-item version of the Health Care Climate Questionnaire modified for adolescents with diabetes and their parents, which measures adolescent and parent perceptions of collaborative decision-making with their diabetes health care professional. Items are rated on a 5-point scale (1=Strongly disagree; 5=Strongly agree). Scores are averaged across items with higher scores indicating higher PCC.
Patient Health Questionnaire for Adolescents (PHQ-A) [53, 54]	An 8-item version* of the PHQ-9 scale, which assesses the presence and severity of depressive symptoms. Respondents rate the frequency with which they have experienced symptoms of depression over the past two weeks on a 4-point scale (0=Not at all; 3=Nearly every day). Items scores are summed to form a total score out of 24. Scores of 5, 10, 15 and 20 represent cut-off points for mild, moderate, moderately-severe and severe depressive symptoms respectively.<br>* the suicide ideation item was omitted.
Problem Areas in Diabetes – Teen (PAID-T) [19]	A 26-item scale that measures the perceived emotional burden of living with diabetes, known as diabetes distress. The PAID-T was adapted from the adult version. Items are rated on a 6-point scale (1=Not a problem; 6=Serious problem). Item scores are summed to form a total score (range 26-156), with higher scores indicating greater diabetes distress.
Problem Areas in Diabetes – Parents of Teens version (P-PAID-T) [29]	The P-PAID-T was adapted from the PAID-T to measure the perceived emotional burden of caring for an adolescent with diabetes. The rating scale, method of scoring and interpretation are the same as for the PAID-T.
WHO-5 Wellbeing Scale [7, 50-52]	A positively-worded 5-item scale that measures general emotional well-being. Each item is scored on a 6-point scale (0=All of the time; 5=At no time). The item scores are summed and multiplied by 4 to form a total score (range 0-100) with higher scores indicating better well-being. A total score <50 suggests impaired emotional well-being.
